# A demographic and clinical panorama of a sixteen-year cohort of soft tissue sarcoma patients in Brazil

**DOI:** 10.1038/s41598-021-02032-5

**Published:** 2021-11-18

**Authors:** Luiza Ohasi de Figueiredo, Augusto Afonso Guerra Júnior, Francisco de Assis Acurcio, Alessandra Maciel Almeida, Mariângela Leal Cherchiglia, Alberto Julius Alves Wainstein, Luiz Claudio Santos Thuler, Angélica Nogueira-Rodrigues

**Affiliations:** 1 Faculty of Medical Sciences of Minas Gerais (FCMMG), Alameda Ezequiel Dias 275, Belo Horizonte, MG 30130-110 Brazil; 2 Hospital Felício Rocho, Av. Do Contorno, 9530 - Barro Preto, Belo Horizonte, MG 30110-934 Brazil; 3 CCATES—Collaborating Centre for Health Technologies Assessment and Excellence in Health, Faculty of Pharmacy - Federal University of Minas Gerais, Av. Antônio Carlos, 6627, Belo Horizonte, 31279-901 Brazil; 4 Faculty of Medicine, Federal University of Minas Gerais, Av. Prof. Alfredo Balena, 190 - Santa Efigênia, Belo Horizonte, MG 30130-100 Brazil; 5 Brazilian National Cancer Institute (INCA), Rio de Janeiro, Praça Cruz Vermelha 23, Rio de Janeiro, RJ 20230-130 Brazil

**Keywords:** Cancer, Surgical oncology

## Abstract

Little is known about soft tissue sarcomas (STS) in Brazil, once the federal statistics regarding estimates on incidence and mortality of the most common cancers that affect the Brazilian population currently do not include STS. This study aims to perform a broad evaluation and description of the epidemiological profile, access to treatment and main clinical outcomes of the Brazilian STS patient. A population-based cohort study of 66,825 patients who underwent procedures related to STS treatment registered in the Brazilian public health system (Sistema Único de Saúde, SUS) databases. Median age was 57 years, 30% of them older than 65 years and 50.7% of the cohort was female. The majority, 50,383 patients (75.4%), was diagnosed between 2008 and 2015. Most prevalent anatomic sites were upper and lower limbs (12.6%) and the registry of sarcomas without a specific location comprehended 29.7% of the cohort. The majority of patients resided in the Northeast (40.2% of the patients). Surgery was the first treatment modality in 77.7% of the cases. For survival analysis, only patients with stage and histological grade information were included. The 1-, 5- and 10-year survival rate of the patients was, respectively, 75.4% (95% CI = 74.1–76.7%), 43.4% (95% CI = 41.5–45.5%) and 18.6% (95% CI = 14.8–23.3%).

## Introduction

Soft-tissue Sarcomas (STS) are a rare and heterogenous group of neoplasms, derived from a myriad of mesodermal (or mesenchymal) cells distributed within the entire body, performing connective roles among over 50 subtypes based on their cell origin and histologic and molecular characteristics, and accounting for less than 1% of all adult solid malignant tumors^1^^.^

The Brazilian National Cancer Institute (INCA) publishes biannually statistics regarding estimates on incidence and mortality of the most common cancers that affect the Brazilian population, STS not included^2^. Few studies about STS have been published in Brazil so far, most of them covering single institutions experiences, none addressing the consolidated Brazilian scenario. The purpose of this study is to perform a broad evaluation and description of the epidemiological profile, access to treatment and main clinical outcomes of the STS patients treated at the Brazilian public health system (Sistema Único de Saúde, SUS).

## Results

Data from 66,852 STS patients registered in the Brazilian national databases (Mortality Information Systems—SIM, Outpatient Information System—SIA/SUS, and Hospital Information System—SIH/SUS from 2000 to 2015 were obtained. These databases include information from public health services from the 26 Brazilian states and the Federal District. This cohort includes patients who underwent at least one of the following three procedures for STS: surgery, chemotherapy or radiotherapy.

Median age was 57 years, 33% of them older than 65 years.50.7% of the cohort (33,940) was female. The majority, 50,383 patients (75.4%), was diagnosed between 2008 and 2015 (Table1). Most prevalent anatomic sites were head and neck (13.6%), followed by upper and lower limbs (12.6%). The registry rate of sarcomas without a determined location or characterized as invasive without a specific location (with ICD 10 identification as C498 and C499) comprehended 29.7% of the cohort (Table 1).

**Table 1:** Epidemiologic Aspects of the Sarcoma Patient in Brazil.

		Soft-tissue Sarcoma
Total		66,852 (100%)
Sex	Female	33,940 (50.7%)
	Male	32,912 (49.3%)
Race	Asian	558 (0.8%)
	White	12,143 (18%)
	Indigenous	9 (0.01%)
	Non-Caucasian (Mixed)	8046 (12%)
	Black	941 (1.5%)
	Undetermined	45,155 (67.6%)
Region	Midwest	3720 (5.6%)
	Northeast	26,885 (40.2%)
	North	2092 (3.1%)
	Southeast	23,378 (35%)
	South	10,777 (16.1%)
Age at diagnosis	18–25	4034 (6%)
	26–35	6308 (9.4%)
	36–45	8619 (13%)
	46–55	12,249 (18.3%)
	56–65	13,591 (20.3%)
	> 65	22,051 (33%)
Year of diagnosis	2000–2003	9170 (13.7%)
	2004–2007	7299 (11%)
	2008–2011	22,957 (34.3%)
	2012–2015	27,426 (41%)
Sarcoma anatomic location	Abdomen and pelvis	3267 (5%)
	Head and neck	15,926 (23.8%)
	Lower limbs	8441 (12.6%)
	Upper limbs	8442 (12.6%)
	Peritoneum and retroperitoneum	6668 (10%)
	Thorax and torso	4230 (6.3%)
	Invasive without specific location	9173 (13.7%)
	Undetermined	10,705 (16%)
Tumor grade at diagnosis	G1	3524 (5.3%)
	G2	635 (1%)
	G3	1629 (2.4%)
	GX	61,064 (91.3%)
Clinical stage at diagnosis	Stage 1	999 (1.5%)
	Stage 2	2917 (4.3%)
	Stage 3	3637 (5.4%)
	Stage 4	6247 (9.3%)
	Undetermined	53,052 (79.5%)
First course-therapy	Limb amputation	625 (1%)
	Lymphadenectomy	388 (0.6%)
	Chemotherapy	9429 (14%)
	Radiotherapy	5481 (8.2%)
	Retroperitoneal resection	3668 (5.5%)
	Wide tumoral excision	26,984 (40.3%)
	Simple tumoral resection	20,277 (30.4%)
Event	Death	17,212 (25.7%)
	Censoring	49,640 (74.3%)

Sub-registration of tumor grade and clinical staging was observed, being GX (undefined) and clinical stage undetermined the most common register for these variables, 91.3% and 79.5%, respectively (Table 1).

The majority of patients resided in the Northeast region (40.2% of the patients) and Southeast (35% of the patients), followed by the South, Midwest and North regions (16.1%, 5.6%, 3.1%, respectively). Other epidemiologic characteristics are summarized in Table 1.

Even though histopathological and molecular data are not available in the databases, it was possible to identify and correlate anatomical location (identified according to the ICD-10) and the choice of initial treatment. From a total of 625 patients who underwent limb amputation, 420 of them (67.2%) had their STSs located in the lower limbs or hips (ICD 10 C49.2), and 157 patients (25.12%) with upper limbs or shoulders compromised by STS (ICD 10 49.1). From the 20,277 patients who underwent local wide excision of their STS, 8734 of them had their tumor located at their head or neck (ICD C49.0), followed by invasive STS without a defined location and unspecified location (C49.9 and C49.8 respectively).

Surgery was the first treatment modality in 77.7% of the cases from the original cohort of 66,852 STS patients, and, focusing on first type of surgical procedure, 70.7% of the patients underwent specific upfront tumoral excision, with 40.3% of the cohort undergoing wide tumoral excisions as first registered treatment, and 30.4% of the cohort undergoing simple tumoral excisions.

Regarding the the combination of surgery, systemic chemotherapy and radiotherapy, as treatment options, the regimen for the STS treatments was diverse(all therapeutic modalities—combined or not—were specified at Table 2). Aproximately 20.7% of the patients were only submitted to clinical treatment with: chemotherapy exclusively (11,38%), radiotherapy exclusively (6,3%) or to a combination of both (3%), without undergoing surgery, during the entire length of the cohort (Table 2). From the 51,942 (77.7% of the original cohort) patients who started their treament by undergoing a surgical procedure, for 48,877 (73.1% of the original cohort) surgery was the only therapy performed.

**Table 2:** Complete Therapeutic Approach of the STS Patient Throughout the Cohort.

	Soft-tissue Sarcoma patients
Total	66,852 (100%)
Surgery only	48,877 (73.1%)
Chemotherapy only	7611 (11.38%)
Radiotherapy only	4246 (6.3%)
Chemotherapy + Radiotherapy	1977 (3%)
Adjuvant Rt	1261 (2%)
Adjuvant Qt	1154 (1.7%)
Adjuvant Qt + Rt	646 (1%)
Neoadjuvant Qt	372 (0.5%)
Neoadjuvant Rt	279 (0.4%)
Neoadjuvant Qt and Adjuvant Qt + Rt	119 (0.2%)
Neoadjuvant Qt and Adjuvant Rt	82 (0.12%)
Neoadjuvant Rt and Adjuvant Qt	68 (0.10%)
Neoadjuvant Qt + Rt	42 (0.06%)
Neoadjuvant Qt + Rt and Adjuvant Qt	46 (0.06%)
Neoadjuvant Rt and Adjuvant Qt + Rt	26 (0.03%)
Neoadjuvant and Adjuvant Qt	17 (0.02%)
Neoadjuvant and Adjuvant Rt	10 (0.01%)
Neoadjuvant and Adjuvant Qt + Rt	10 (0.01%)
Neoadjuvant Qt + Rt and Adjuvant Rt	9 (0.01%)

Qt: chemotherapy; Rt: radiotherapy.

**Figure1:**
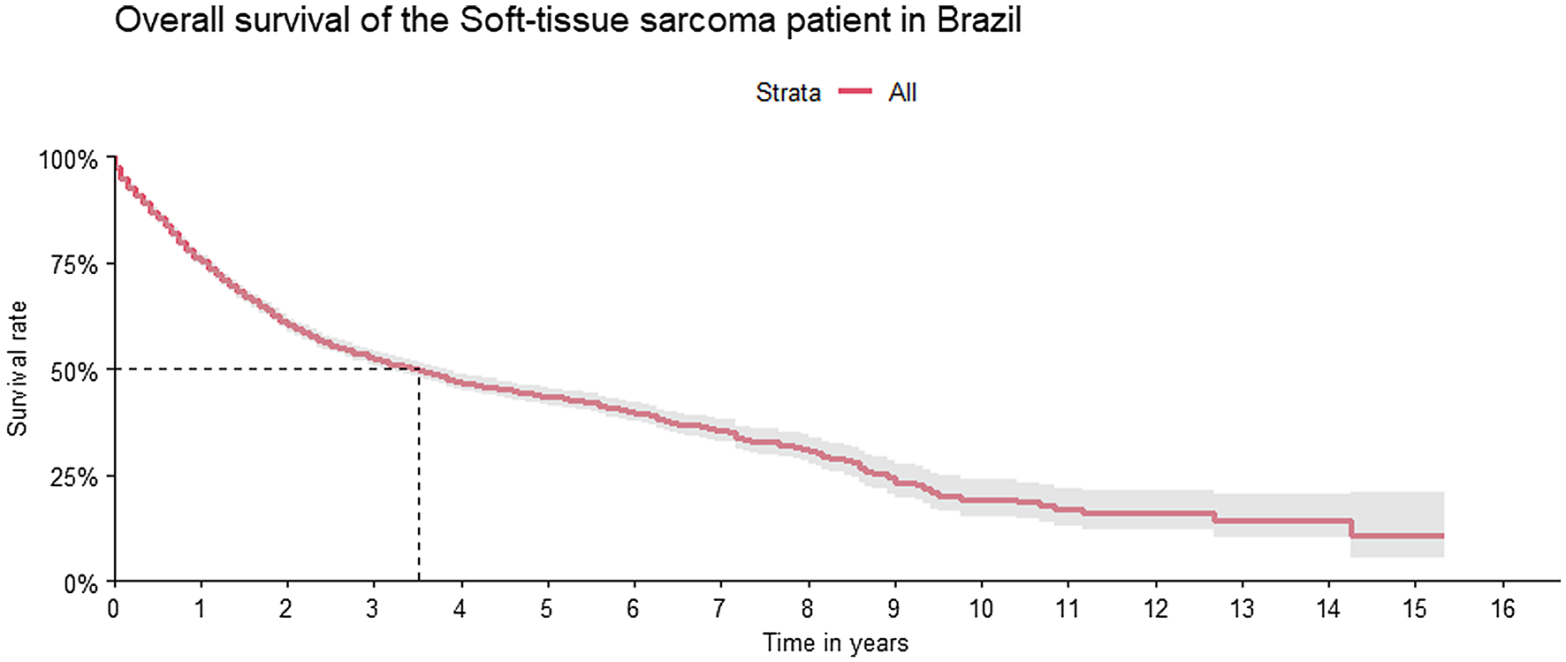
Overall Survival of the Soft-tissue Sarcoma Patient in Brazil (only staged and graded patients).

For survival analysis, a sub-cohort was stablished including only patients with complete staging and grading data, and after exclusion of head and neck patients. In this sub-cohort 4980 patients were evaluated and 1,992 events (deaths) were observed. The median overall survival was 42 months (38–46 months 95% CI), approximately 3.5 years, as shown on Fig. 1.

**Figure2:**
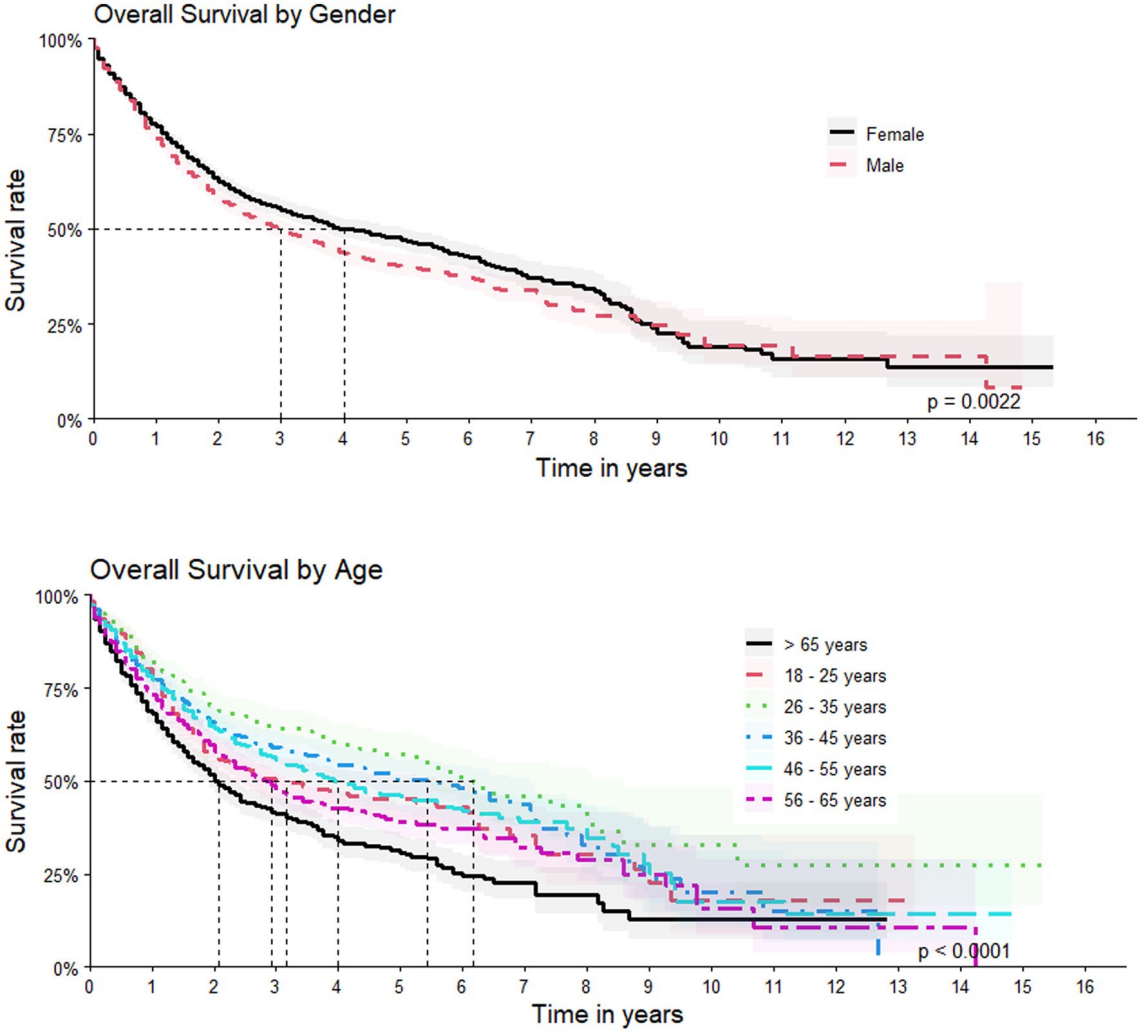
Overall survival by gender and age.

The 1-, 5- and 10-year survival rate of the patients was, respectively, 75.4% (95% CI = 74.1–76.7%), 43.4% (95% CI = 41.5–45.5%) and 18.6% (95% CI = 14.8–23.3%). Survival rate was lower amongst men, with 1-, 5- and 10-year survival rate of, respectively 73.7% (95% CI = 71.8–75.6%), 40.2% (95% CI = 37.4–43.1%) and 19.4% (95% CI = 14.01–26.8%), as well as in elderly patients (> 65 years of age) with 1- and 5-year survival rate of, respectively, 68.0% (95% CI = 65.01–71.2%) and 30.6% (95% CI = 26.64%–35.2%) (Fig. 2).

The following characteristics were associated to lower survival rates in univariate analysis of the subcohort: male gender (HR = 1.147; 95% CI = 1.051–1.253); age above 65 years old (HR = 1.515; 95% CI = 1.369–1.677); tumor of undetermined locations (HR = 1.318; CI = 1.194–1.456) ;clinical stage 4 (HR = 2.102; CI = 1.922–2.299) at the moment of first treatment, patients first treated with chemotherapy (HR = 1.591; CI = 1.456–1.738)), as first theraputical approach (Table 3).

**Table 3:** Univariate analysis of sub cohort: patient and tumor characteristics impact on survival.

		HR (95% IC)	*p* value
Total			
Sex	Male	1.147 (1.051–1.253)	*p* = < 0.005
Race	Asian	0.7363 (0.4739–1.144)	*p* = 0.2
	White	1.04 (0.9528–1.136)	*p* = 0.4
	Indigenous	–	–
	Non–Caucasian (Mixed)	0.9716 (0.8789–1.074)	*p* = 0.6
	Black	1.006 (0.7988–1.1267)	*p* = 1
	Undetermined	0.9952 (0.8871–1.117)	*p* = 0.9
Region	Midwest	1.099 (0.9033–1.336)	*p* = 0.4
	Northeast	0.868 (0.7793–0.9668)	*p* = 0.009
	North	0.8487 (0.646–1.115)	*p* = 0.2
	Southeast	1.056 (0.9674–1.153)	*p* = 0.2
	South	1.068 (0.9596–1.19)	*p* = 0.2
Age at diagnosis	18–25	0.9851 (0.8477–1.145)	*p* = 0.8
	26–35	0.6559 (0.5663–0.7596)	*p* = < 0.005
	36–45	0.8193 (0.7231–0.9284)	*p* = < 0.005
	46–55	0.8895 (0.7952–0.9949)	*p* = 0.04
	56–65	1.137 (1.023–1.264)	*p* = 0.02
	> 65	1.515 (1.369–1.677)	*p* = < 0.005
Year of diagnosis	2000–2003	0.2843 (0.1934–0.4179)	*p* = < 0.005
	2004–2007	0.8997 (0.7704–1.051)	*p* = 0.2
	2008–2011	1.103 (1.007–1.208)	*p* = 0.03
	2012–2015	1.067 (0.9675–1.177)	*p* = 0.2
Anatomic location of the sarcoma	Abdomen and pelvis	1.137 (0.9662–1.337)	*p* = 0.1
	Lower limbs	0.7963 (0.7179–0.8833)	*p* = < 0.005
	Upper limbs	0.837 (0.7376–0.9498)	*p* = 0.005
	Peritoneum and retroperitoneum	1.128 (1.001–1.272)	*p* = 0.05
	Thorax and torso	0.9476 (0.7975–1.126)	*p* = 0.5
	Invasive without specific location	0.8839 (0.7138–1.095)	*p* = 0.2
	Undetermined	1.318 (1.194–1.456)	*p* = < 0.005
Tumor grade at diagnosis	G1	0.8759 (0.8011–0.9577)	*p* = < 0.005
	G2	1.169 (1.018–1.342)	*p* = 0.03
	G3	1.085 (0.9853–1.194)	*p* = 0.1
Clinical stage at diagnosis	Stage 1	0.488 (0.3917–0.6079)	*p* = < 0.005
	Stage 2	0.4775 (0.4189–0.5443)	*p* = < 0.005
	Stage 3	0.8353 (0.7541–0.9252)	*p* = < 0.005
	Stage 4	2.102 (1.922–2.299)	*p* = < 0.005
First course–therapy	Limb amputation	1.419 (1.02–1.974)	*p* = 0.04
	Lymphadenectomy	2.676 (0.3767–19.02	*p* = 0.4
	Chemotherapy	1.591 (1.456–1.738)	*p* = < 0.005
	Radiotherapy	0.9216 (0.8276–1.026)	*p* = 0.1
	Retroperitoneal resection	0.7835 (0.6294–0.9754)	*p* = 0.02
	Wide tumoral excision	0.5687 (0.4885–0.6619)	*p* = < 0.005
	Simple tumoral resection	0.6617 (0.563–0.7778)	*p* = < 0.005

*p* value determined by Score (logrank) test and value refers to the comparison between groups.

HR: hazard ratio; CI: confidence interval.

**Figure 3:**
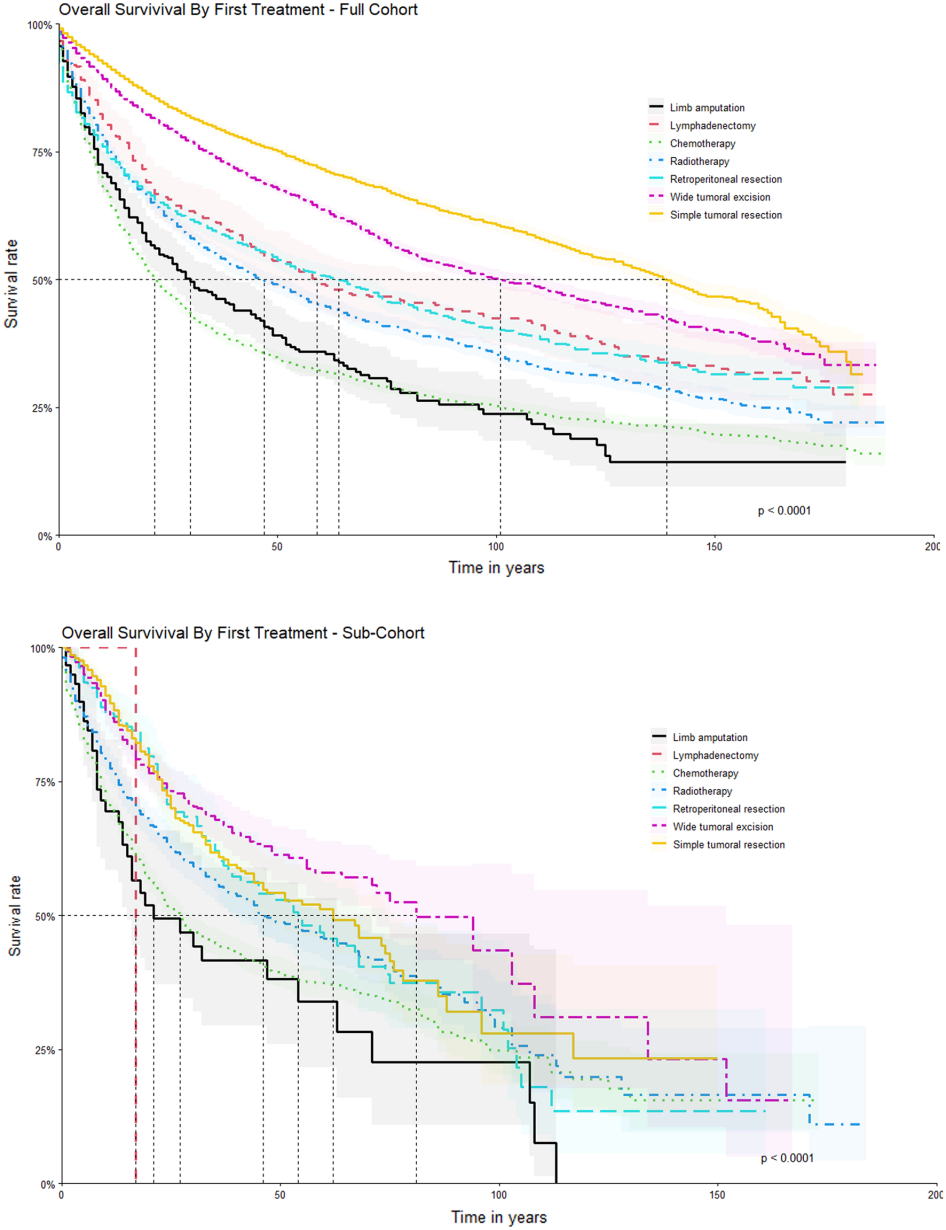
Compared Survival by Type of First Treatment between Full Cohort and Only Staged Patients (Above: Full cohort with 66,852 patients. Below: Sub-cohort with 4980 staged patients).

Different survival rates, mainly within the groups of patients who underwent limb amputation, lymphadenectomy and retroperitoneal resection, were also observed as shown in the Kaplan–Meier curves below, after the exclusion of the non-staged and graded patients from the survival analysis (Fig. 3).

**Figure 4:**
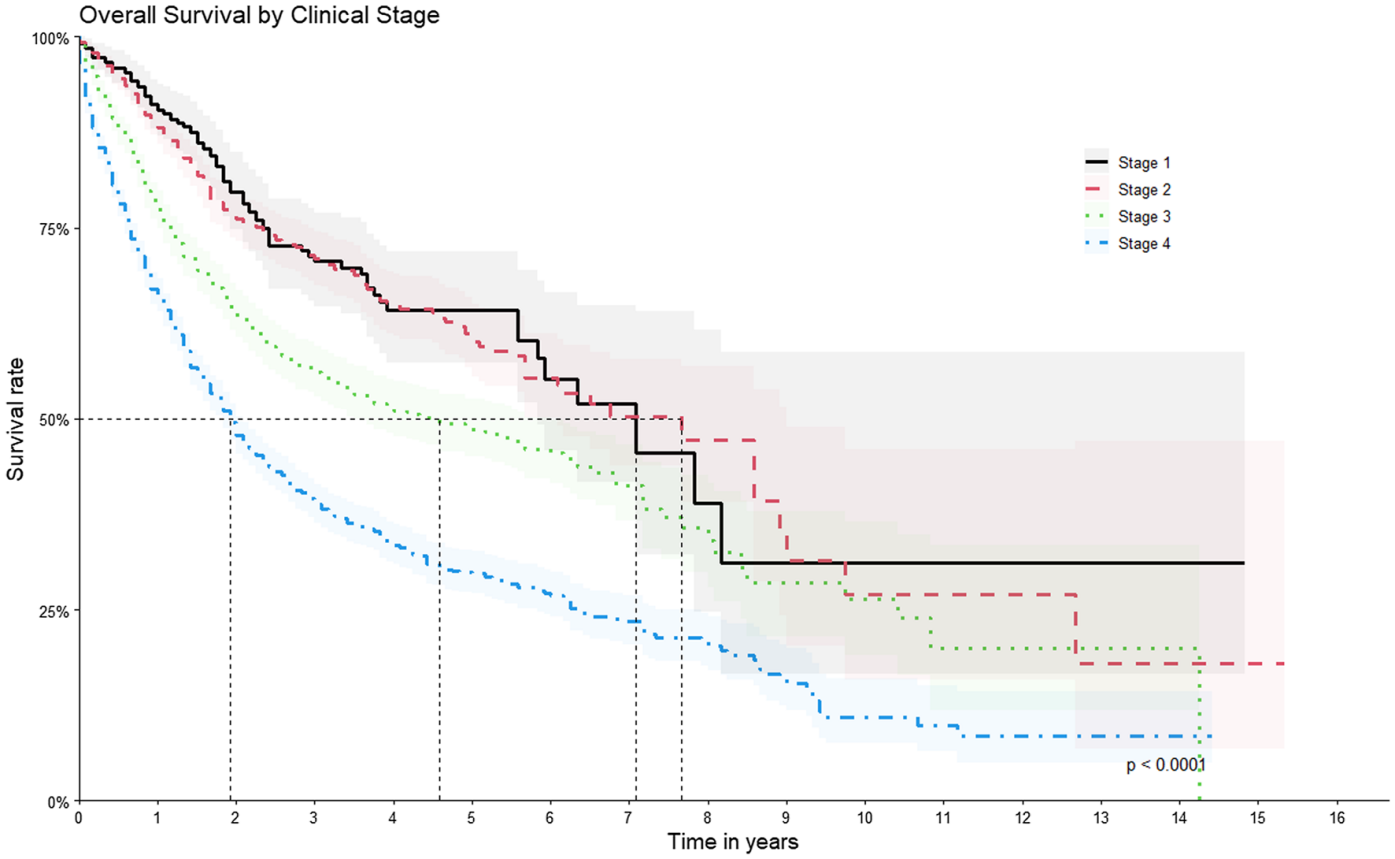
Overall Survival by Clinical Stage.

Regarding patients’ survival rates according to each specific clinical stage in the sensitivity analysis of the sub-cohort, the following 1-, 5- and 10-year values were observed, approximately: stage 1 with 90.4% (95% CI = 87–93.8%), 60.2% (95% CI = 52.1–69.5%), respectively and no data regarding 10-year survival; stage 2 with 88.1% (95% CI = 85.99–90.3%), 60% (95% CI = 55.5–65.0%), and 27% (95% CI = 15.79–46.1%), respectively; stage 3 with 77.3% (95% CI = 74.9–79.8%), 48.7% (95% CI = 45.1–52.6%), and 26.3% (95% CI = 19–36.4%), respectively, and stage 4 with 66.16% (95% CI = 64.09–68.3%), 29.73% (95% CI = 27.1–32.6%), and 10.95% (95% CI = 7.52–16%), respectively (Fig. 4).

## Discussion

Including 66,852 patients, this is the largest study with epidemiological and clinical aspects of STS in Brazil, with data from all states of the five Brazilian regions and covering a long time-frame (16 years). Some international studies have been conducted by compiling health-care generated data in order to better know the STS patient profile and to acknowledge the impact of this disease all over the world, such as Saltus, in 2018^3^ in Germany, and Burningham, in 2012^1^ in the United States. Brazil has more than 210 million inhabitants, a highly admixed population and peculiar access to health care, since more than 70% of the Brazilian population count on its public health services for cancer care. Exploring potentiality of available Brazilian data to explore epidemiology STS profile is fundamental for governmental planning, health technologies and health economics assessment, and it may help other LMICs by mirroring their realities.

Epidemiologically, there was an almost even distribution between genres, with a slight majority of the patients being female (50.7%), aged above 65 years old (33%), of white race (18.1%) and living in the Northeast region (40,2%). However, male gender aged above 65 years old and with STS of undetermined locations (according to ICD-10 classification) showed worst prognostic predictors. The epidemiological profile of the Brazilian STS patient showed to be similar to the ones described by other international cohorts. In Germany, Saltus^3^ describes their patients as evenly distributed between males and females, aged above 65 years old and with tumors located most commonly in the lower extremity, trunk and head and neck. In Canada, Bozzo^4^ described the STS population with a slight tendency to male (1.5:1 ratio male to female) and aged above 50 years old in 68% of the cases. Regarding the American population of STS patients, the AJCC described the STS patients as with a tendency to male (53.7%), with STS mostly located on lower extremities (57.5%) and with a mean age of 59 years old^5^.

Regarding the STS localization results, the large number of head and neck STS patients was cautiously analyzed regarding epidemiological characteristics and were removed from the survival analyses. As mentioned, the anatomical location was identified and determined according to the ICD-10, and it is relevant to highlight that “Malignant neoplasm of connective and soft tissue of head, face and neck” and “Malignant neoplasm of other connective and soft tissue” have a slight similar coding, (respectively C49.0 and C49), and the original SIM and SIA registry requires a 3 digit ICD-10 code, which may have mistakenly increased the notification under “head and neck” anatomic location by the erroneous addition of an extra 0 during the digitalization of official forms. This analysis is corroborated after the assessment of the correlation of procedures performed according to each specific anatomic location (Table 4). The option to maintain a broad spectrum of STS, except head and neck STS due to misclassification, regardless of the different standard treatment is justified for the epidemiologic relevance of profiling de Brazilian STS patient, in general.

**Table 4:** Distribution of procedures based on anatomic location.

Anatomic location type of procedure	C49.4 + C49.5	C49.0	C49.8	C49.9	C49.2	C49.1	Any C48	C49.3 + C49.6	TOTAL
Limb amputation	4	4	4	17	420	157	16	3	625
Lymphadenectomy	10	55	19	140	10	10	117	27	388
Chemotherapy	742	1132	324	1931	1706	1410	1342	842	9429
Radiotherapy	505	1147	183	1157	1225	506	278	480	5481
Retroperitoneal resection	2	2	3	5			3656		3668
Wide tumoral excision	1240	8734	4094	6651	1792	1587	1185	1701	26,984
Simple tumoral resection	764	4852	4546	804	3288	4772	74	1177	20,277
TOTAL	3267	15,926	9173	10,705	8441	8442	6668	4230	**66,852**

When first course treatment was analyzed, patients who underwent specific resection of their tumors had a better prognosis (Wide tumoral excision, HR = 0.5687, 95% CI = 0.4885–0.6619) and Simple tumoral resection with an HR = 0.6617, 95% CI = 0.563–0.7778) ) and, according to the Kaplan–Meier survival analysis (Fig. 3), had a significant better survival rate than the other treatment modalities, with initial lymphadenectomy and limb amputation being the least performed procedures. These results go in accordance with the standard treatment of the STS, where an adequate (and wide if necessary) resection is indicated, with a predilection of limb-sparring surgeries, since 1985^6,7^^.^

Regarding the use of chemotherapy and radiotherapy on the sarcoma patient, literature indicates the use of radiotherapy, currently, in neoadjuvant or in adjuvant settings in order de improve local control rate and decrease the recurrence rate (mainly in extremity STS), but has no improvement in overall survival. The ESMO recommendation favors adjuvant radiotherapy when tumors size is above 5 cm or deeply located or high grade and when not R0 resection occurs and NCCN recommends neoadjuvant radiation therapy with external beam for microscopic residual disease control and adjuvant treatment when insufficient margins, with local control rates of 95% with preoperative RT and negative margins, but reinforces that radiotherapy is not substitute for the definitive surgical resection^8,9^. The use of chemotherapy, both neoadjuvant or adjuvant, remains controversial with benefits described as the facilitation of surgery, when used perioperatively, or in a systemic modality, with anthracyclines with ifosfamides for high risk patients with thoracic or extremity STS^8^.

Treatment wise, the Brazilian are, apparently, in resonance with European and American practices. When we correlate the data on clinical staging, type of first course procedure and combination of clinical and surgical approaches, we observed that the vast majority of patients who underwent only chemotherapy were, in its majority, clinically staged as 4. Neoadjuvant radiotherapy or neoadjuvant with adjuvant radiotherapy were mostly administered in patients staged 2 or 3. If we consider that the non-surgical treatment is reserved for patients with no possibility of surgical intervention, our study had 13,834 who underwent chemotherapy and radiotherapy exclusively or combined, with different proportions of clinical staging distributions, and not only to higher stages. It is important to reinforce that many different histological subtypes of STS have different recommendation of systemic treatment.

When comparing survival curves with another countries, it was observed a small difference in 1-year overall survival between the Brazilian an American STS patient. A further analysis of the survival by age shows a worst prognosis when long time survival is evaluated amongst men. After the first year of diagnosis, around 84% of male and 84.5% of female STS patients in United States are expected to have survived, compared to 73.7% and 77%, respectively, in Brazil, with the latter demonstrating a slight better result within the female population. The 5-year and 10-year survival data, however, show a rapid decrease in survival of patients from both countries. The worst results are seen with the Brazilian male population with, respectively 40.2% and 19.4% 5- and 10-year survival rates, compared to 64,9% and 59,2% 5- and 10-year survival rates of the American male population. And, regardless of a better overall outcome regarding survival risks, the female population still has a decrease of survival rates amongst American (with 66.5% and 61.1% 5- and 10-year survival rates, respectively) and Brazilian women (46.6% and 18.2% 5- and 10-year survival rates, respectively). The SEERS database collects cancer incidence data from population-based cancer registries covering approximately 34.6 percent of the U.S. population, with an STS incidence valued less than 6 cases for 100,000. However, the annual incidence of the STS in Brazilian population remains unknown, and was not calculated in this study due to this cohort not being a representation of all sarcoma patients registered on the SUS databases (due to exclusion criteria) or the totality the patients treated for STS in Brazil, as a whole.

By comparing STS data from different populations, it was observed that British patient’s survival chance is slightly lower than the Brazilian ones, and 9.2% lower when compared to American population within the first year of STS follow up (1-year survival rates of 75%, 82% and 84.2% respectively). And this order of, potentially, improved outcomes of the Brazilian patient shifts when we compare the rates of 5-year survival data available (65.6% in United States, 55% in United Kingdom and 43.4% in Brazil). Still, it is possible to identify a maintenance in decrease of survival comparison when we observe the 10-year survival rates, United Kingdom presenting a better survival rate (45%) than Brazil (18.6%) amongst the STS patients. The values for both countries remain, however, significantly lower than American 10-year survival rate, which reaches 60.1%. The progressive decrease in survival of the Brazilian STS patients might also be related to the fact that the majority of the evaluated patients underwent first treatment for their tumor after 65 years of age, with 33% of this cohort being constituted of elderly patients^10–12^.

This study is based on the gathering of information obtained from mandatory forms filled out by health care providers and provide the data for administrative databases. As in the many cohorts, several limitations related to the use of a secondary database can be found in this study, such as inconsistency, inadequacy of collected information, confounding factors and missing information. The difficulties to access this data reinforces the necessity of a unified platform. On account of the coverage of this study and its development based on databases generated by the performance of oncologic treatment for STS (surgical, systemic—with chemotherapy—or loco regional—with radiotherapy), information that is present on medical charts and medical files generated by standard follow up consultations were not available and neither these patients could be accurately identified for this investigation, after the elaboration of the cohort. The Brazilian government is currently working on a unified platform.

A major limitation of this study is the use of an administrative database, and failures in filling out clinical information, coding procedures challenges, absence of socioeconomic and demographic variables. Attention to underreporting and inadequate data filling must be paid, considering that the lack of direct access to histopathological, molecular, clinical and therapeutic variables or information of the sarcoma subtypes, made the diagnosis confirmation possible only by the registry of the patient under C48, C48.0, C48.1, C48.2, C48.8, C49, C49.0, C49.1, C49.2, C49.3, C49.4, C49.5, C49.6, C49.8. C49.9, according to the tenth revision of the international classification of diseases (ICD-10), topographic localization associated with the therapeutic utilized (and depending on if the patient underwent systemic treatment) the only mean to infer over the possible subtype of the mass/tumor registered, and the significant number of missing data. The lack of staging and grading data from these patients must also be mentioned, taking into account that the treatment modality recommended for patients and prognosis relies on this information. The lack of information regarding histological grading and clinical staging of a large number of patients (classified as “GX” and “undetermined clinical staging”) is presumed to occur because of sub registration. Due to the large number of patients treated with only surgery (without complementation with chemotherapy or radiotherapy) the filing of SIA forms, in which staging and tumor grading related information is mandatory, was—most likely—not performed. After correlation with all the clinical and surgical treatments analyzed, it is believed that the majority of them underwent simple resection or wide resection, not going through other treatment modalities afterwards. The high percentage of missing data on Brazilian national cancer databases have already been reported by other authors^13^, and it should be carefully reformed to enable trustworthy epidemiological analysis in the country. Despite these limitations this is the largest cohort of STS in Brazil so far, and consolidated epidemiological data is essential to better structure STS control programs in the country.

## Methods

**Figure 5:**
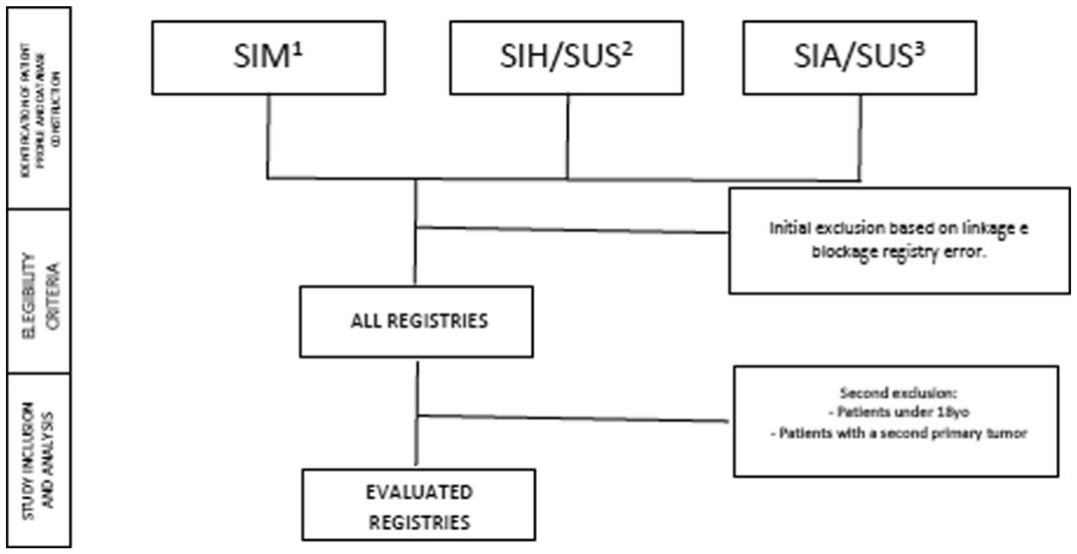
Study design. ^1^Mortality Information Systems (SIM), ^2^Outpatient Information System (SIA/SUS), ^3^Hospital Information System (SIH/SUS).

We conducted a retrospective cohort study of patients who underwent hospital and outpatient clinical and surgical procedures related to STS treatment in SUS from 01/01/2000 to 12/31/2015. A National Database of Health centered on the individual was built through a deterministic-probabilistic record linkage of three administrative databases: The Outpatient Information System (SIA/SUS), the Hospital Information System (SIH/SUS), and the Mortality Information System (SIM) (Fig. 5). The construction and validation of this database has been described and validated elsewhere^13,14^, and it has been used in previous research studies published. The time period of the cohort was determined based on the data available within the database^14–18^.

The study included patients with STS according to the tenth revision of the international classification of diseases (ICD-10) (oncologic diagnostic identified as C48, C48.0, C48.1, C48.2, C48.8, C49, C49.0, C49.1, C49.2, C49.3, C49.4, C49.5, C49.6, C49.8. or C49.9), submitted to any type of surgery or systemic treatment (chemotherapy and/or radiotherapy) as first course therapy related to their disease from 2000 to 2016. The first registry of surgery, systemic treatment (chemotherapy), or radiotherapy in association with a respective STS ICD-10 was considered T0 (“time zero”; baseline). The event used for survival analysis was death registered by the Mortality Information Systems (SIM) (mandatory for all Brazilian population) and right censoring was defined as not observing/having registered death until December 2016.

We analyzed the factors that influenced survival using univariate analysis for each descriptive variable and evaluated their association the event—death. The Kaplan–Meier method was used to estimate the cumulative probability of survival. The different survival curves were compared using the logrank test. The hazard ratio (HR) for progression to the event was calculated by univariate analyses considering a 95% confidence interval (95% CI) and using the Cox proportional hazards model and comparing each variable with the values of the same group.

The following variables were analyzed: age at diagnosis, country´s region, sex, diagnosis´ year, tumor anatomic location (identified according to the tenth revision of the international classification of diseases—ICD-10), tumor grade at diagnosis(5, 19), clinical stage at diagnosis (Stage I: tumor is small and low grade -GX or G1;Stage II: tumor is small and G2 or G3; Stage III: Tumor is larger and G2 or G3; and Stage IV: cancer has spread to other parts of the body), (any G)(5, 19)), first course-therapy (surgery, chemotherapy, radiotherapy) and time of death. For survival analysis, a sub-cohort was stablished including only patients with complete staging and grading data, and after exclusion of head and neck patients.

### Statistical analysis

Descriptive statistical analysis of all variables in this study was performed: frequency distribution for categorical variables and central tendency for continuous variables.

The Kaplan–Meier method was used to estimate the cumulative probability of survival and different survival curves were compared using the log-rank test. The hazard-ratio (HR) for progression to the event was calculated by univariate analysis considering a 95% confidence interval.

The management of the data and statistical analysis was performed using ‘R’ Version 1.3.1056 (R Foundation for Statistical Computing) and MySQL,19 version 5.0(Oracle Corporation), considering a significance level of 5%.

### Ethical aspects

This study was approved by the Faculdade de Ciências Médicas de Minas Gerais (FCMMG) internal review board (IRB) in Belo Horizonte, Brazil and registered under de protocol number: CEP 3.928.928/2020–2. The research was conducted in accordance with all relevant Brazilian guidelines and regulations and followed the criteria of the Declaration of Helsinki.

Informed consent was waived by the ethics committee IRB of the Faculdade de Ciências Médicas de Minas Gerais (FCMMG) once the study was based on variables obtained by the confluence of a secondary publicly available SUS database, without any identification of the patients available from the original source and containing no information that can lead to direct identification of the 66,852 patients.

## Conclusion

Including 66,852 patients treated at the Brazilian public health system from 2000 to 2015, this is the largest cohort of STS in the country. Male gender, age above 65 years old and with STS of the peritoneum and retroperitoneum, thorax and torso, were identified as negative prognostic predictors. In a sub-cohort including fully staged and graded subjects, 1, 5 and 10 y survivals were: 82%, 57% and 42%, in line with international data. Much is yet to be understood about STS sarcoma patient in Brazil and other LMICs, and comprehensive patient data registration and unified platforms are essential.

## Data Availability

The datasets generated during and/or analyzed during the current study are available from the corresponding author on reasonable request.
